# Supplementary material for: Monocytes Count, NLR, MLR and PLR in Canine Inflammatory Bowel Disease
Source: Animals (Basel). 2024 Mar 8;14(6):837. doi: 10.3390/ani14060837 (PMC10967434; doi:10.3390/ani14060837)
Supplement: Supplementary file 1 [file animals-14-00837-s001.zip › animals-2896433-supplementary.pdf]

Figure S1: Receiver-operating characteristics (ROC) curve for the most relevant variables.

A. ROC curve illustrating red blood cells (RBC) concentrations ($10^6/\mu L$) that distinguish clinically healthy dogs from dogs with Inflammatory Bowel Disease (IBD).

B. ROC curve illustrating hemoglobin (Hb) concentrations (g/dL) that distinguish clinically healthy dogs from dogs with IBD.

C. ROC curve illustrating white blood cells (WBC) concentrations ($10^3/\mu L$) that distinguish clinically healthy dogs from dogs with IBD.

D. ROC curve illustrating neutrophils concentrations ($10^3/\mu L$) that distinguish clinically healthy dogs from dogs with IBD.

E. ROC curve illustrating monocytes concentrations ($10^2/\mu L$) that distinguish clinically healthy dogs from dogs with IBD.

F. ROC curve illustrating neutrophil-to-lymphocyte (NLR) ratio that distinguish clinically healthy dogs from dogs with IBD.

G. ROC curve illustrating monocyte-to-lymphocyte (MLR) ratio that distinguish clinically healthy dogs from dogs with IBD.

H. ROC curve illustrating platelet-to-lymphocyte (PLR) ratio that distinguish clinically healthy dogs from dogs with IBD.

A.

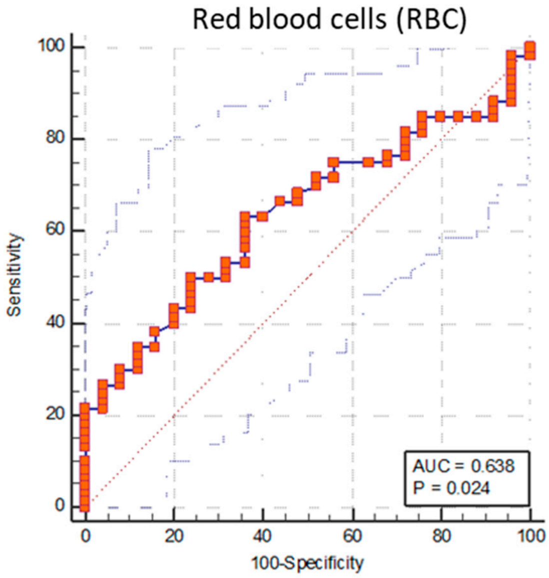

B.

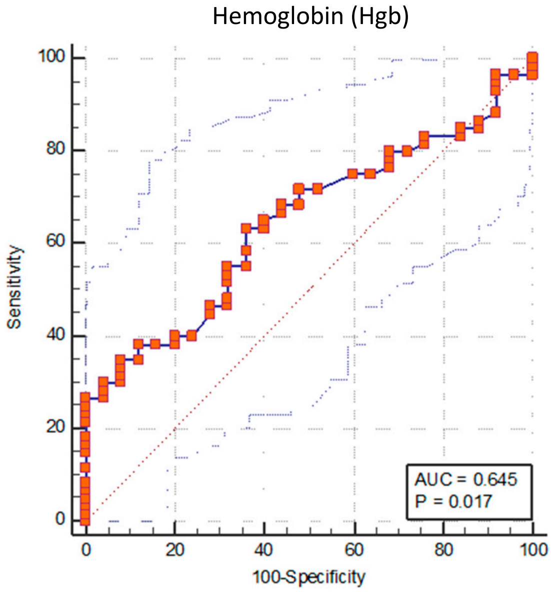

C.

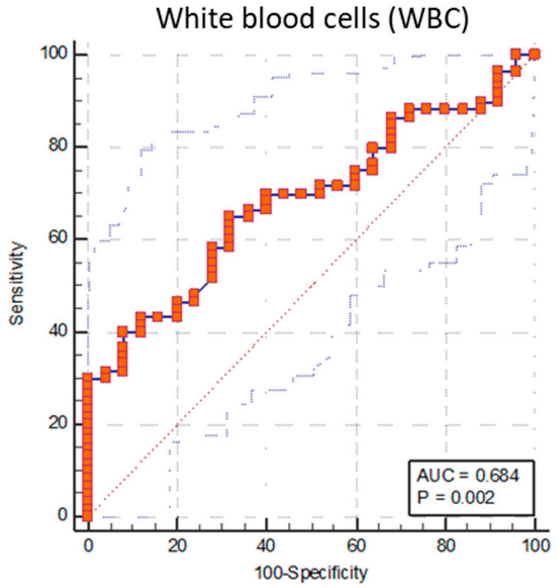
White blood cells (WBC)
Sensitivity
100-Specificity
AUC = 0.684
P = 0.002

D.

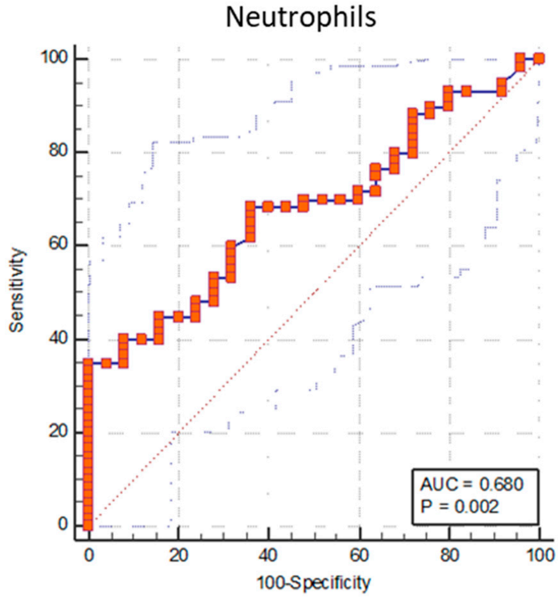
Neutrophils
Sensitivity
100-Specificity
AUC = 0.680
P = 0.002

E.

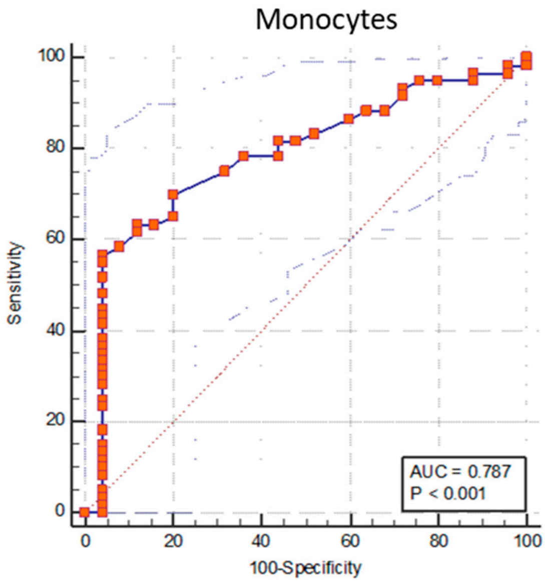
Monocytes
Sensitivity
100-Specificity
AUC = 0.787
P < 0.001

F.

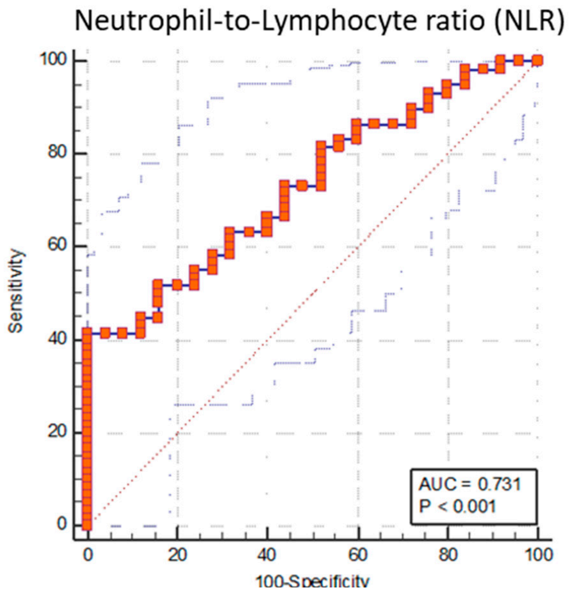
Neutrophil-to-Lymphocyte ratio (NLR)
Sensitivity
100-Specificity
AUC = 0.731
P < 0.001

G.

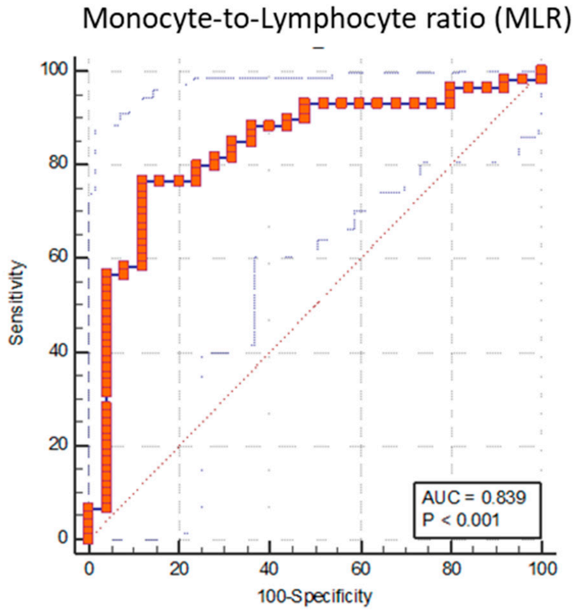
Monocyte-to-Lymphocyte ratio (MLR)
Sensitivity
100-Specificity
AUC = 0.839
P < 0.001

H.

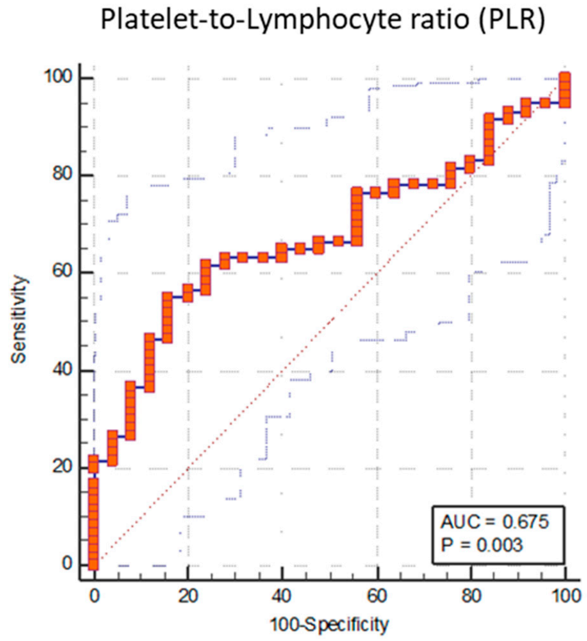
Platelet-to-Lymphocyte ratio (PLR)
Sensitivity
100-Specificity
AUC = 0.675
P = 0.003
